# Computational Approaches for Integrative Analysis of the Metabolome and Microbiome

**DOI:** 10.3390/metabo7040062

**Published:** 2017-11-18

**Authors:** Jasmine Chong, Jianguo Xia

**Affiliations:** 1 Institute of Parasitology, McGill University, Montreal, QC H3A 0G4, Canada; jasmine.chong@mail.mcgill.ca; 2 Department of Animal Science, McGill University, Montreal, QC H3A 0G4, Canada

**Keywords:** metabolome, microbiome, multi-omics integration, integrative analysis

## Abstract

The study of the microbiome, the totality of all microbes inhabiting the host or an environmental niche, has experienced exponential growth over the past few years. The microbiome contributes functional genes and metabolites, and is an important factor for maintaining health. In this context, metabolomics is increasingly applied to complement sequencing-based approaches (marker genes or shotgun metagenomics) to enable resolution of microbiome-conferred functionalities associated with health. However, analyzing the resulting multi-omics data remains a significant challenge in current microbiome studies. In this review, we provide an overview of different computational approaches that have been used in recent years for integrative analysis of metabolome and microbiome data, ranging from statistical correlation analysis to metabolic network-based modeling approaches. Throughout the process, we strive to present a unified conceptual framework for multi-omics integration and interpretation, as well as point out potential future directions.

## 1. Introduction

The human gut microbiome is a complex biological system that performs several vital functions for the host, such as digestion and degradation of macromolecules, production of vitamins, and training of the host immune system. Over the millenniums, humans have coevolved symbiotically with these gut microbes. They interact with each other primarily through evolutionarily conserved chemical dialogs that involve different metabolites and pathways [1,2]. The increasingly affordable high-throughput sequencing technologies have enabled invaluable insights into the structure and functional potential of the microbiome [3,4,5]. For instance, metagenomics studies of the gut microbiome have shown that community structures shift with dietary changes, and that metabolic potentials are different between obese and lean mice [6,7]. While promising, key limitations of these approaches (i.e., marker gene sequencing or metagenomics) are their inability to directly measure functional activity and to identify microbial traits associated with functions. Downstream omics technologies that analyze the transcriptome (metatranscriptomics), proteome (metaproteomics) and metabolome (metabolomics) have shown that significant genes identified through metagenomics might not necessarily be expressed [8,9].

Of the omics technologies, metabolomics plays a key role in connecting host phenotype and microbiome function [10,11]. Metabolomics is the systematic study of all small molecules within a biological system. Unlike other meta-omics, metabolites and metabolic pathways are relatively conserved across species. The gut metabolome consists of metabolites produced from both the host and the microbial community. Coupling metabolomics with metagenomics has great potential to shift current microbiome research towards understanding community functions and interactions with the host. Much work has been done to analyze individual omics data, with many powerful bioinformatics tools developed over the past decade to enable metabolome and microbiome profiling [12,13,14,15,16,17]. In comparison, analyzing multi-omics data is still in its infancy, usually requiring development and application of advanced statistical algorithms to leverage multiple heterogeneous yet interconnected data matrices. In this review, we focus specifically on recent progress and applications of different computational strategies for integration of metabolomics in the context of microbiome studies.

## 2. Metabolomics Data Integration in the Microbiome

**Figure 1:**
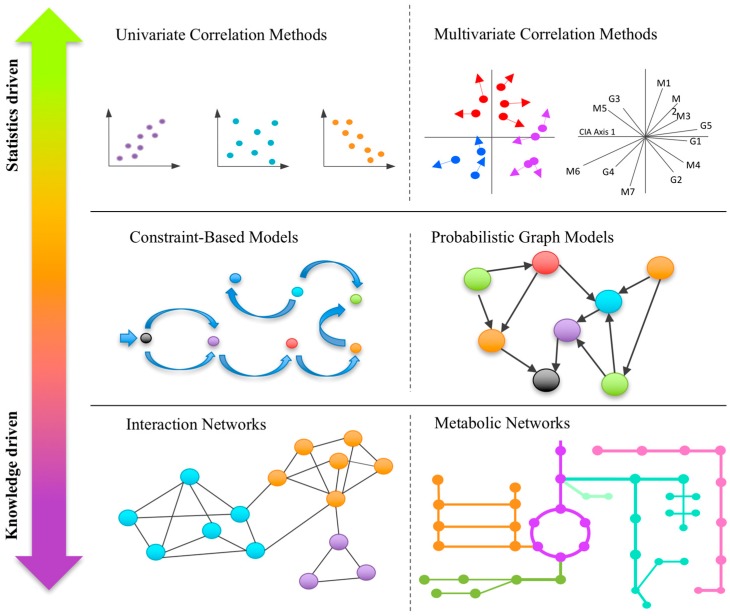
A graphical summary of the major types of computational approaches for integrative analysis of multi-omics data. These methods range from purely statistical approaches (**top**) to primarily knowledge-driven approaches (**bottom**). Integrating these two approaches may offer great potential for future development in the field.

Multi-omics data consists of two or more matrices that share the same sample IDs but contain different biological variables such as genes, metabolites, or operational taxonomic units (OTUs). Based on whether they take into consideration prior knowledge, these methods range from predominantly statistics-based to knowledge-driven integrations (Figure 1). Statistical integration employs univariate or multivariate analysis to understand correlations between biological variables in different omics layers, while knowledge-based approaches aim to understand potential mechanistic links by projecting the significant biological variables identified from individual omics layers into an existing knowledge base, often represented as interaction networks (such as metabolic networks). More recent developments aim to directly incorporate prior knowledge into statistical models to improve both statistical power and interpretability of the results.

### 2.1. Univariate Correlation-Based Approaches

The simplest approaches for omics integration are univariate correlation analysis to determine if there are strong linear relationships (Pearson’s correlation) or monotonic relationships (Spearman’s correlation) between individual metabolites (metabolome) and genes or taxa (microbiome). For instance, Theriot et al. performed Spearman correlation analysis between the mouse gut microbiome and metabolome to identify relationships between metabolite–OTU pairs [18]. Further unsupervised clustering analysis of these correlations revealed that these metabolite–OTU pairs tend to group together according to different environmental states [18]. In a multi-omics study of the goat rumen microbiome, Mao et al. applied univariate correlation methods to create a Pearson’s correlation matrix between genera and metabolites [19]. The authors identified a clear correlation between altered community structure of the rumen microbiome and changes in metabolite profiles with increasing carbohydrate intake [19].

While performing univariate correlations is relatively straightforward, these methods suffer from a high rate of false positives, requiring researchers to control the type I error rate by performing multiple testing corrections. Also, while associations between metabolites and the microbiome can be extrapolated, these associations often lack context for interpretation in terms of biological plausibility and mechanistic insight. Univariate correlation-based approaches are often used together with other knowledge-based approaches to aid in data interpretation.

### 2.2. Multivariate Correlation-Based Approaches

Although significantly more complex than univariate methods, multivariate approaches can simultaneously consider interactions between and within data matrices. Due to the high-dimensional nature of omics data, dimension-reduction methods have become the predominate methods to perform statistical integration. For the remainder of this section, consider two data matrixes, X (*n* × *p*) and Y (*n* × *q*), where *n* represent the same individuals or samples, and *p* and *q* represent different sets of omics variables.

Dimension-reduction techniques aim to reduce large number of variables into a handful of new components or principal variables, with minimal loss of information. Multivariate methods for omics integration are usually extensions of commonly used dimension reduction techniques, including principal component analysis (PCA) and partial least squares (PLS). PCA is a data-reduction technique that identifies linear combinations of variables that maximizes the variance within one data matrix (X). PLS is a supervised method aiming to maximize the covariance between extracted components of X and Y. Conventional PLS requires users to define X and Y as response or predictive variables, and does not consider inherent systematic variation that may exist within each dataset that does not correspond with the outcome [20]. An extension of PLS, two-way orthogonal PLS (O2–PLS), disregards matrix assignment and treats X and Y as equals. The O2–PLS separates the variation in each dataset into three blocks, joint, unique and residual, and is then able to identify significantly predictive features of the joint variation [20,21,22]. For instance, El-Aidy et al. used O2–PLS for pair-wise integration of metabolomic, transcriptomic and metagenomic data of germ-free mice undergoing colonization of normal mice gut-microbiota [23]. The authors identified strong correlations between early microbial colonizers and variations in urine metabolites, as well as correlations between colonic tissue metabolites and the upregulation of genes involved in *O*- and *N*-glycan biosynthesis and degradation [23].

Canonical correlation analysis (CCA) [24] and co-inertia analysis (CIA) [25] are two other multivariate correlation methods often employed for omics integration. CCA is a feature-extraction method that identifies the best linear combination of X and Y that maximizes the correlation between components. To perform CCA, variables within the data set should be linearly independent, and the number of samples should not be less than the number of variables. Both assumptions are usually not met in omics data. To address this limitation, sparse variations of CCA have been developed including sparse CCA (sCCA) [26], kernel CCA [27] and a regularized version of CCA (RCCA) [28]. Kostic et al. used a sparse version of CCA to integrate the gut microbiome and the gut metabolome of infants predisposed to type 1 diabetes (T1D) [29]. The authors identified a canonical variable that consisted of increased *Ruminococcus* and decreased *Veillonella* abundances that correlated significantly with increased Sphingomyelin and decreased lithocholic acid levels [29]. The authors suggested that these T1D-associated bacteria promote a pro-inflammatory microbiome through such metabolites that permits the pathogenesis of T1D [29].

Co-inertia analysis (CIA) [25] was initially used in ecological studies and has since been used for omics integration. It describes the co-structure between two datasets by maximizing the covariance between components. CIA first performs a data-reduction technique such as PCA or correspondence analysis on X and Y separately, and then constrains the resulting components so that the squared covariances between X and Y are maximized. In a simpler sense, CIA can be considered as a PCA of the joint covariances of X and Y [30]. Hill et al. used CIA to examine the relationship between urinary metabolites and metagenomic data from the gut microbiome in pre-term and full-term infants [31]. The study identified significant covariance between the metabolomic and metagenomic data, with distinct separation between the pre-term infants from the full-term infants [31]. Liu et al. also used CIA to evaluate the covariance between the metabolites and genes of obese and lean humans [32]. The covariance between the genes and metabolites showed clear separation patterns between these two groups, and microbes differentially enriched were associated with tyrosine, phenylalanine, glutamate and branched-chain amino acids [32].

Procrustes analysis (PA) is a statistical technique that utilizes data-reduction methods such as PCA and CCA for visual integration of omics data [33]. PA is a fast and simple visualization technique that superimposes the principal components of two datasets at the low-dimensional space and allows researchers to quickly examine the congruency of their multi-omics datasets. For example, McHardy et al. [34] performed PA between metabolome and microbiome data to determine the strength of the inter-omics relationship, and showed that the microbiome and metabolome were more similar in the cecum compared to the sigmoid. To investigate the roles of fermented food exposure in humans [35], Quinn et al. also applied PA on 16S rRNA microbiome data and metabolomics data to gain an integrated look at the relationship between the microbiome and metabolome. PA on its own is not sufficient to draw strong conclusions, but can be complimented with other multivariate methods such as CCA [35].

The aforementioned multivariate methods, much like univariate methods, are biologically naïve, often resulting in biologically improbable solutions. For instance, changes to the different molecular layers of the microbiome often do not occur simultaneously, and integration of prior knowledge to account for such time-scale differences could help remedy this issue. For instance, Garali et al. [36] recently introduced the regularized generalized CCA (RGCCA) and its sparse counterpart (SRGCCA) as a multi-block approach for integrative exploratory analysis of multi-omics data. Through the use of a design matrix, shrinkage parameter and scheme function, SRGCCA/RGCCA encompasses several known omics integration techniques such as PCA, PLS, CCA and their own extensions as special cases. Importantly, this method allows for the incorporation of prior knowledge through the design matrix, which takes into account block connections [36].

## 3. Knowledge-Based Approaches for Omics Integration

This approach leverages our existing knowledge framework about relationships between metabolites, species and/or genes to integrate different omics data. This information can be collected from public databases, through literature mining or by computational predictions. Our knowledge about the communications between microbial species and their interactions with their host can be intuitively represented as networks in which metabolites/microbes/genes are presented as nodes and their known/predicted interactions as edges. Such network representation allows for both intuitive visual exploration and topological analysis to identify important links or key actors within the microbial community. One commonly used measure is node degree, which measures the number of connections between a node and its surrounding nodes. Highly connected nodes (also known as hubs) tend to have greater influence upon the network [37]. Another useful measure is modularity, which is defined as a group of nodes that are more densely connected with each other as compared to nodes outside of that group. These nodes are considered more functionally related within the context. For instance, Greenblum et al. revealed that the gut microbiomes of obese humans are less modular than their lean counterparts [38]. Topological approaches together with visualization are frequently used to highlight key differences between different disease states, and to suggest potential biological mechanisms. 

### 3.1. Correlation/Interaction-Based Community Networks

The simplest method for knowledge-based omics integration is the correlation network, which is created based on pair-wise relationships between biological entities measured in the omics data. The pair-wise relationships can be computed directly from the omics data itself or based on third-party resources. For instance, McHardy et al. [34] used pair-wise Spearman correlation between microbiome and metabolome data to construct an interaction network of the cecum and sigmoid. Correlations less than *q* ≥ 0.2 were removed from the network, and edges were colored based on a positive or negative correlation. A more biologically motivated approach is to connect two nodes based upon (potential) shared biological interaction, such as the known/predicted positive or negative impacts of microbial growth between microbial species based upon potential cross-feeding, collaboration or competition. For instance, Sung et al. created a metabolite transport network of the gut microbiota based upon an extensive literature review of the metabolites and macromolecules 567 gut microbiota can import or export [39]. Using this information, positive or negative pair-wise associations between the microbiota were calculated and visualized to create a community interaction network [39]. Both approaches have been successful at identifying novel associations between omics data. However, because they focus solely upon pair-wise relationships between nodes, they may miss complex interactions within the microbiome data [40].

### 3.2. Metabolic Networks

While correlation-based network reconstructions touch upon microbial species interaction, they are unable to provide more detailed mechanistic information about these interactions. Metabolic models, which are comprehensive metabolic reconstructions of an organism, are an alternative to the previous interaction-based network approach. These models can be used as a scaffold for omics data integration, whereby they provide key mechanistic details surrounding microbiome function and activity. More specifically, genome-scale metabolic models (GEMs) are complete metabolic maps of an organism, containing the entire set of metabolic reactions and permitting integration of metabolomics and metagenomics data in a more biologically meaningful context. While construction of GEMs is time-consuming, several well-annotated GEMs have been produced for a variety of organisms including human, mice and recently, human gut microbiome species [41,42,43]. These GEMs can be merged to create community-wide metabolic networks, and further linked with tissue-specific host GEMs to help investigate host–microbiome interactions [44,45].

#### 3.2.1. Topological Analysis of Metabolic Models

The seed set framework [46] is one commonly used topological-based approach for GEM investigations. This framework applies a graph theory-based algorithm to identify the minimal subset of exogenously derived compounds required to produce all other compounds within a metabolic network. Greenblum et al. used the seed set framework in the context of a community gut microbiome metabolic network, and found that enzymes identified in the seed set were overrepresented in obese and IBD-associated enzymes [38]. The seed set framework has also been applied to predict microbe–microbe interactions by computing competition and mutualism scores based upon the predicted seed set of each microbe–microbe pair at the genus level [47]. Competition scores were based upon the overlap of seed set compounds, and mutualism scores were based upon the overlap of compounds that a microbe can consume from which another microbe can provide. This method can be further extended through the prediction of microbial interactions at the species/strain level, such as with the recently published resource of GEMs of the human gut microbiota [43].

Topological analysis could be used to perform gene/reaction knockouts within in-silico community metabolic network reconstructions [48]. In this case, omics can easily be integrated to create state-specific metabolic reconstructions, with the overlay of measured genes/metabolites to determine the presence or absence of reactions. Graph-based algorithms could then be applied to identify the effect of removing certain genes/metabolites/reactions, such as the loss of functions or the decreased production of compounds. This novel topological-based method could identify potential biomarkers or key genes/metabolites to be targeted for future in-vitro experiments.

#### 3.2.2. Community Metabolic Models

Predicted relative metabolic turnover (PRMT) is a network-based modeling method that was initially created to predict community metabolic function solely from metagenomic information. It uses metagenomic information such as the relative abundance and average enzyme function counts per phyla to infer the metabolic potential of each phyla, which is then used to model the community metabolome via calculated PRMT scores [49]. This method was first used to investigate the role of microbial communities in the Western English Channel [49]. Using PRMT, the authors accurately predicted the metabolic turnover of microbial communities with seasonal changes, and identified bacteria that were highly correlated with the consumption or production of certain metabolites [49]. More recently, PRMT has been extended to include metabolomics data to correlate changes in microbial communities with changes in the measured community metabolome (MIMOSA) [50]. The authors adapted the PRMT method to use estimated gene abundances from metagenomic data to produce community-wide metabolic potential (CMP) scores for each metabolite and sample. These CMP scores represent the relative capacity of community members to produce or consume metabolites based upon a priori metabolic information for each species such as from GEMs or KEGG. The authors then compared variations in calculated CMP scores to variations in measured metabolite levels. They used this information to estimate well-predicted metabolites, whose predicted CMP scores are sufficiently explained by the metabolic potential of the microbiome community. Using the CMP scores for each metabolite, they also estimated key species and genes required for their production [50]. This method could be further enhanced through the inclusion of host dynamics, gene expression information to create more accurate CMP scores, and inclusion of reversible reactions to aid metabolite prediction.

## 4. Summary and Future Perspectives

In this review, we have discussed statistical approaches and knowledge-based frameworks for integrating metabolomics into current microbiome data analysis and interpretation. Currently, researchers need to have a deep understanding and programming skills to use multivariate statistical methods, which represent significant barriers for their wider applications. Easy-to-use bioinformatics tools are urgently needed to address this gap. Similarly, it is far more pragmatic to focus on metabolic network topology and connectivity patterns to analyze the microbiome. The use of prior biological knowledge to reconstruct networks is one of the most intuitive methods, giving appropriate context for data interpretation. This approach does have its limitations, however; because it is based upon existing network knowledge, it does not identify de-novo relationships.

There are two promising approaches that show great potential for integration of the metabolome and microbiome. The first are probabilistic graph models such as Bayesian network (BN) models, which are popular knowledge-based network approaches. BNs are graphical models that represent probabilistic causal relationships between variables, and aim to identify the most probabilistic network that is predictive of the observed data. Bayesian networks were recently used to model the succession of bacterial colonization in the infant gut [51], and Zhu et al. [52] integrated metabolomics and transcriptomics data to create probabilistic causal network models of cell regulation in yeast. BN models of the microbiome integrated with multiple omics are promising though have yet to be achieved. Constraint-based modeling is the second method for metabolomics and microbiome integration that performs simulations of the microbiome. A well-known constraint-based modeling method is flux balance analysis (FBA), which simulates the flow of metabolites within a biological system [53]. Through the inclusion of many parameters such as enzyme kinetics, reaction fluxes and stoichiometry, FBA can be used to simulate microbial growth or predict metabolite production. Multispecies constraint-based models are however still in their infancy, and are limited to understanding interactions between only a few microbes [54,55,56,57]. Multispecies models are further complicated by determining the correct compartmentalization of species within a biological system, as well as choosing an optimal community objective function. The amount of knowledge required to perform constraint-based modeling on such a large and complex scale such as the gut microbiome is unrealistic at the current stage. Modeling the microbiome using this method would be computationally intensive, thereby limiting the utility of this method to small-scale community network reconstructions.

The aim of this review is to raise awareness of the current computational approaches for metabolomics integration with microbiome data. Statistical correlation-based and biological network-based approaches for omics integration have been discussed together with their application examples and potential limitations. A multifaceted approach that utilizes several approaches that complement one another may help gain deeper insights into microbiome function. Furthermore, integration of prior knowledge will ground results into a more realistic environment and reduce false positives. Bioinformatics tools that integrate statistics, visualization and biological knowledge in a user-friendly interface will greatly facilitate integration of metabolomics within current microbiome studies.

## References

[1] Martin F.-P.J., Sprenger N., Yap I.K., Wang Y., Bibiloni R., Rochat F., Rezzi S., Cherbut C., Kochhar S., Lindon J.C. (2009). Panorganismal gut microbiome—Host metabolic crosstalk. J. Proteome Res..

[2] Candela M., Guidotti M., Fabbri A., Brigidi P., Franceschi C., Fiorentini C. (2011). Human intestinal microbiota: Cross-talk with the host and its potential role in colorectal cancer. Crit. Rev. Microbiol..

[3] Gill S.R., Pop M., DeBoy R.T., Eckburg P.B., Turnbaugh P.J., Samuel B.S., Gordon J.I., Relman D.A., Fraser-Liggett C.M., Nelson K.E. (2006). Metagenomic analysis of the human distal gut microbiome. Science.

[4] Consortium H.M.P. (2012). Structure, function and diversity of the healthy human microbiome. Nature.

[5] Yatsunenko T., Rey F.E., Manary M.J., Trehan I., Dominguez-Bello M.G., Contreras M., Magris M., Hidalgo G., Baldassano R.N., Anokhin A.P. (2012). Human gut microbiome viewed across age and geography. Nature.

[6] Turnbaugh P.J., Ridaura V.K., Faith J.J., Rey F.E., Knight R., Gordon J.I. (2009). The effect of diet on the human gut microbiome: A metagenomic analysis in humanized gnotobiotic mice. Sci. Transl. Med..

[7] Turnbaugh P.J., Ley R.E., Mahowald M.A., Magrini V., Mardis E.R., Gordon J.I. (2006). An obesity-associated gut microbiome with increased capacity for energy harvest. Nature.

[8] Franzosa E.A., Morgan X.C., Segata N., Waldron L., Reyes J., Earl A.M., Giannoukos G., Boylan M.R., Ciulla D., Gevers D. (2014). Relating the metatranscriptome and metagenome of the human gut. Proc. Natl. Acad. Sci. USA.

[9] Verberkmoes N.C., Russell A.L., Shah M., Godzik A., Rosenquist M., Halfvarsson J., Lefsrud M.G., Apajalahti J., Tysk C., Hettich R.L. (2008). Shotgun Metaproteomics of the Human Distal Gut Microbiota.

[10] Fiehn O. (2002). Metabolomics—The link between genotypes and phenotypes. Functional Genomics.

[11] Patti G.J., Yanes O., Siuzdak G. (2012). Innovation: Metabolomics: The apogee of the omics trilogy. Nat. Rev. Mol. Cell Biol..

[12] Dhariwal A., Chong J., Habib S., King I.L., Agellon L.B., Xia J. (2017). Microbiomeanalyst: A web-based tool for comprehensive statistical, visual and meta-analysis of microbiome data. Nucleic Acids Res..

[13] Xia J., Mandal R., Sinelnikov I.V., Broadhurst D., Wishart D.S. (2012). Metaboanalyst 2.0—A comprehensive server for metabolomic data analysis. Nucleic Acids Res..

[14] Xia J., Psychogios N., Young N., Wishart D.S. (2009). Metaboanalyst: A web server for metabolomic data analysis and interpretation. Nucleic Acids Res..

[15] Xia J., Sinelnikov I.V., Han B., Wishart D.S. (2015). Metaboanalyst 3.0—Making metabolomics more meaningful. Nucleic Acids Res..

[16] Schloss P.D., Westcott S.L., Ryabin T., Hall J.R., Hartmann M., Hollister E.B., Lesniewski R.A., Oakley B.B., Parks D.H., Robinson C.J. (2009). Introducing mothur: Open-source, platform-independent, community-supported software for describing and comparing microbial communities. Appl. Environ. Microbiol..

[17] Caporaso J.G., Kuczynski J., Stombaugh J., Bittinger K., Bushman F.D., Costello E.K., Fierer N., Peña A.G., Goodrich J.K., Gordon J.I. (2010). Qiime allows analysis of high-throughput community sequencing data. Nat. Methods.

[18] Theriot C.M., Koenigsknecht M.J., Carlson P.E., Hatton G.E., Nelson A.M., Li B., Huffnagle G.B., Li J., Young V.B. (2014). Antibiotic-induced shifts in the mouse gut microbiome and metabolome increase susceptibility to clostridium difficile infection. Nat. Commun..

[19] Mao S.Y., Huo W.J., Zhu W.Y. (2016). Microbiome–metabolome analysis reveals unhealthy alterations in the composition and metabolism of ruminal microbiota with increasing dietary grain in a goat model. Environ. Microbiol..

[20] Trygg J., Wold S. (2003). O2-pls, a two-block (x–y) latent variable regression (LVR) method with an integral OSC filter. J. Chemom..

[21] Bylesjö M., Eriksson D., Kusano M., Moritz T., Trygg J. (2007). Data integration in plant biology: The O2PLS method for combined modeling of transcript and metabolite data. Plant J..

[22] El Bouhaddani S., Houwing-Duistermaat J., Salo P., Perola M., Jongbloed G., Uh H.-W. (2016). Evaluation of o2pls in Omics data integration. BMC Bioinformatics.

[23] El Aidy S., Derrien M., Merrifield C.A., Levenez F., Doré J., Boekschoten M.V., Dekker J., Holmes E., Zoetendal E.G., Van Baarlen P. (2013). Gut bacteria–host metabolic interplay during conventionalisation of the mouse germfree colon. ISME J..

[24] Hotelling H. (1936). Relations between two sets of variates. Biometrika.

[25] Dolédec S., Chessel D. (1994). Co-inertia analysis: An alternative method for studying species–environment relationships. Freshw. Biol..

[26] Lin D., Zhang J., Li J., Calhoun V.D., Deng H.-W., Wang Y.-P. (2013). Group sparse canonical correlation analysis for genomic data integration. BMC Bioinform..

[27] Yamanishi Y., Vert J.-P., Kanehisa M. (2004). Protein network inference from multiple genomic data: A supervised approach. Bioinformatics.

[28] De Bie T., De Moor B. (2003). On the regularization of canonical correlation analysis. Int. Sympos. ICA BSS.

[29] Kostic A.D., Gevers D., Siljander H., Vatanen T., Hyötyläinen T., Hämäläinen A.-M., Peet A., Tillmann V., Pöhö P., Mattila I. (2015). The dynamics of the human infant gut microbiome in development and in progression toward type 1 diabetes. Cell Host Microbe.

[30] Thioulouse J. (2011). Simultaneous analysis of a sequence of paired ecological tables: A comparison of several methods. Ann. Appl. Stat..

[31] Hill C.J., Lynch D.B., Murphy K., Ulaszewska M., Jeffery I.B., O’Shea C.A., Watkins C., Dempsey E., Mattivi F., Tuohy K. (2017). Evolution of gut microbiota composition from birth to 24 weeks in the infantmet cohort. Microbiome.

[32] Liu R., Hong J., Xu X., Feng Q., Zhang D., Gu Y., Shi J., Zhao S., Liu W., Wang X. (2017). Gut microbiome and serum metabolome alterations in obesity and after weight-loss intervention. Nat. Med..

[33] Gower J.C. (1975). Generalized procrustes analysis. Psychometrika.

[34] McHardy I.H., Goudarzi M., Tong M., Ruegger P.M., Schwager E., Weger J.R., Graeber T.G., Sonnenburg J.L., Horvath S., Huttenhower C. (2013). Integrative analysis of the microbiome and metabolome of the human intestinal mucosal surface reveals exquisite inter-relationships. Microbiome.

[35] Quinn R.A., Navas-Molina J.A., Hyde E.R., Song S.J., Vázquez-Baeza Y., Humphrey G., Gaffney J., Minich J.J., Melnik A.V., Herschend J. (2016). From sample to multi-omics conclusions in under 48 hours. mSystems.

[36] Garali I., Adanyeguh I.M., Ichou F., Perlbarg V., Seyer A., Colsch B., Moszer I., Guillemot V., Durr A., Mochel F. (2017). A strategy for multimodal data integration: Application to biomarkers identification in spinocerebellar ataxia. Brief. Bioinform..

[37] Agler M.T., Ruhe J., Kroll S., Morhenn C., Kim S.-T., Weigel D., Kemen E.M. (2016). Microbial hub taxa link host and abiotic factors to plant microbiome variation. PLoS Biol..

[38] Greenblum S., Turnbaugh P.J., Borenstein E. (2012). Metagenomic systems biology of the human gut microbiome reveals topological shifts associated with obesity and inflammatory bowel disease. Proc. Natl. Acad. Sci. USA.

[39] Sung J., Kim S., Cabatbat J.J.T., Jang S., Jin Y.-S., Jung G.Y., Chia N., Kim P.-J. (2017). Global metabolic interaction network of the human gut microbiota for context-specific community-scale analysis. arXiv.

[40] Faust K., Sathirapongsasuti J.F., Izard J., Segata N., Gevers D., Raes J., Huttenhower C. (2012). Microbial co-occurrence relationships in the human microbiome. PLoS Comput. Biol..

[41] Sigurdsson M.I., Jamshidi N., Steingrimsson E., Thiele I., Palsson B.Ø. (2010). A detailed genome-wide reconstruction of mouse metabolism based on human recon 1. BMC Syst. Biol..

[42] Thiele I., Swainston N., Fleming R.M., Hoppe A., Sahoo S., Aurich M.K., Haraldsdottir H., Mo M.L., Rolfsson O., Stobbe M.D. (2013). A community-driven global reconstruction of human metabolism. Nat. Biotechnol..

[43] Magnúsdóttir S., Heinken A., Kutt L., Ravcheev D.A., Bauer E., Noronha A., Greenhalgh K., Jäger C., Baginska J., Wilmes P. (2017). Generation of genome-scale metabolic reconstructions for 773 members of the human gut microbiota. Nat. Biotechnol..

[44] Wang Y., Eddy J.A., Price N.D. (2012). Reconstruction of genome-scale metabolic models for 126 human tissues using mcadre. BMC Syst. Biol..

[45] Agren R., Bordel S., Mardinoglu A., Pornputtapong N., Nookaew I., Nielsen J. (2012). Reconstruction of genome-scale active metabolic networks for 69 human cell types and 16 cancer types using init. PLoS Comput. Biol..

[46] Borenstein E., Kupiec M., Feldman M.W., Ruppin E. (2008). Large-scale reconstruction and phylogenetic analysis of metabolic environments. Proc. Natl. Acad. Sci. USA.

[47] Steinway S.N., Biggs M.B., Loughran T.P., Papin J.A., Albert R. (2015). Inference of network dynamics and metabolic interactions in the gut microbiome. PLoS Comput. Biol..

[48] Zhang C., Hua Q. (2016). Applications of genome-scale metabolic models in biotechnology and systems medicine. Front. Physiol..

[49] Larsen P.E., Collart F.R., Field D., Meyer F., Keegan K.P., Henry C.S., McGrath J., Quinn J., Gilbert J.A. (2011). Predicted relative metabolomic turnover (PRMT): Determining metabolic turnover from a coastal marine metagenomic dataset. Microb. Inform. Exp..

[50] Noecker C., Eng A., Srinivasan S., Theriot C.M., Young V.B., Jansson J.K., Fredricks D.N., Borenstein E. (2016). Metabolic model-based integration of microbiome taxonomic and metabolomic profiles elucidates mechanistic links between ecological and metabolic variation. mSystems.

[51] McGeachie M.J., Sordillo J.E., Gibson T., Weinstock G.M., Liu Y.-Y., Gold D.R., Weiss S.T., Litonjua A. (2016). Longitudinal prediction of the infant gut microbiome with dynamic bayesian networks. Sci. Rep..

[52] Zhu J., Sova P., Xu Q., Dombek K.M., Xu E.Y., Vu H., Tu Z., Brem R.B., Bumgarner R.E., Schadt E.E. (2012). Stitching together multiple data dimensions reveals interacting metabolomic and transcriptomic networks that modulate cell regulation. PLoS Biol..

[53] Orth J.D., Thiele I., Palsson B.Ø. (2010). What is flux balance analysis?. Nat. Biotechnol..

[54] Shoaie S., Karlsson F., Mardinoglu A., Nookaew I., Bordel S., Nielsen J. (2013). Understanding the interactions between bacteria in the human gut through metabolic modeling. Sci. Rep..

[55] El-Semman I.E., Karlsson F.H., Shoaie S., Nookaew I., Soliman T.H., Nielsen J. (2014). Genome-scale metabolic reconstructions of *Bifidobacterium* adolescentis L2–32 and *Faecalibacterium* prausnitzii A2–165 and their interaction. BMC Syst. Biol..

[56] Shoaie S., Ghaffari P., Kovatcheva-Datchary P., Mardinoglu A., Sen P., Pujos-Guillot E., de Wouters T., Juste C., Rizkalla S., Chilloux J. (2015). Quantifying diet-induced metabolic changes of the human gut microbiome. Cell Metab..

[57] Harcombe W.R., Riehl W.J., Dukovski I., Granger B.R., Betts A., Lang A.H., Bonilla G., Kar A., Leiby N., Mehta P. (2014). Metabolic resource allocation in individual microbes determines ecosystem interactions and spatial dynamics. Cell Rep..

